# The Intersectionality of Gender and Wealth in Adolescent Health and Behavioral Outcomes in Brazil: The 1993 Pelotas Birth Cohort

**DOI:** 10.1016/j.jadohealth.2019.08.029

**Published:** 2020-01

**Authors:** Romina Buffarini, Safa Abdalla, Ann M. Weber, Janaína Calu Costa, Ana Maria B. Menezes, Helen Gonçalves, Fernando Cesar Wehrmeister, Valerie Meausoone, Gary L. Darmstadt, Cesar G. Victora

**Affiliations:** a Postgraduate Program in Epidemiology, Federal University of Pelotas, Pelotas, Rio Grande do Sul, Brazil; b Department of Pediatrics, Stanford University School of Medicine, Stanford, California; c Center for Population Health Sciences, Stanford University School of Medicine, Palo Alto, California

**Keywords:** Overweight, Smoking, Violence, Happiness, Sadness, Mental health, Socioeconomic factors, Gender identity, Sex, Adolescent

## Abstract

**Purpose:**

Brazilian society is characterized by deep socioeconomic inequalities. Using data from a population-based birth cohort, we explored how the intersectionality of family income and gender may affect adolescent health and behavioral outcomes.

**Methods:**

Children born in 1993 in the Brazilian city of Pelotas have been followed up prospectively at the age of 15 years when the follow-up rate was 85.7% of the original cohort. Participants answered standardized questionnaires, and anthropometric measures were obtained. Outcomes in five domains were studied: overweight (body mass index above +1 SD of the World Health Organization standard for age and sex), cigarette smoking (in the previous month), violence (fight in which someone was injured, in the past year), self-reported unhappiness (based on a face scale), and psychological symptoms (Strengths and Difficulties Questionnaire). Monthly family income was recoded in quintiles.

**Results:**

Results were available for more than 4,101–4,334 adolescents, depending on the outcome. Overweight was more common among boys than girls (29.7% and 25.6%; *p* = .004) and was directly related to family income among boys (*p* < .001), but not among girls (*p* = .681). Smoking was less common among boys than girls (12.3% and 21.0%, *p* < .001) and showed strong inverse association with income among girls (*p* < .001), but not among boys (*p* = .099). Reported violence was twice as common among boys than girls (16.4% vs. 8.0%; *p* < .001); an inverse association with income was present among girls (*p* < .001), but not for boys (*p* = .925). Boys and girls were similarly likely to report being unhappy (18.4% and 20.1%; *p* = .176), with an inverse association with family income in girls. Psychological symptoms were slightly less common among boys than girls (25.3% and 28.3%; *p* = .014), with strong inverse associations with income in both sexes (*p* < .001). Adolescent girls from poor families were the group with the highest prevalence for three of the five outcomes: smoking, unhappiness, and psychological problems.

**Conclusions:**

Gender norms influence adolescent health and behavioral outcomes, but the direction and strength of the associations are modified by socioeconomic position. Preventive strategies must take into account the intersectionality of gender and wealth.

**Implications and Contribution**

In a population affected by deep social divides, these findings show that gender interacts with socioeconomic position in determining risks to adolescent health because of behavioral conditions including overweight, smoking, violence, happiness, and mental health. Comparisons of male and female adolescents should take into account their socioeconomic stratification at societal level.

During adolescence, the health and behaviors of boys and girls are impacted by societal rules and influences—norms—that go well beyond biological sex-specific vulnerabilities. In particular, the adoption of behaviors typically associated with being masculine or feminine exerts a major influence on health-related conditions that adolescent boys and girls will carry into later life [[1], [2], [3], [4], [5], [6]].

Gender norms can thus influence the choices adolescents make and shape their identities. Health-related gender norms or their manifestations, therefore, may vary according to social standing of their families and neighborhoods [7], such that differences between boys and girls may depend on their position in the social structure within a given society.

Brazil, as an upper-middle-income country, is facing rapid demographic and health transitions. Noncommunicable diseases and violence now constitute the leading causes of death in the country, whereas mental illness remains a major cause of morbidity [8]. Although some risk factors such as tobacco smoking are becoming less common, others such as overweight and obesity are rapidly increasing [9]. A national study in large cities across the country showed that among school-going adolescents aged 12–17 years, 5.7% smoked at the time of the interview, and 8.4% were obese [10,11]. These characteristics could intersect with gender norms and influence health throughout the life course [5].

Brazilian society is characterized by deep, long-standing social inequalities, documented in a large body of national literature linking poverty to poor health outcomes. Despite important progress in the reduction of poverty and improvements in health care since the 1990s [12], there is evidence of a reversal in recent years as a consequence of poor economic growth and government austerity [13].

Studies of intersectionality move beyond examining individual risk factors such as sex or income and focus on how such factors interact to determine how health is shaped across population groups in different contexts [14]. Such studies are highly important because they capture the complexity of the processes through which social status affects health and how these processes are altered by other factors such as gender. More importantly, such studies can help to generate hypotheses about the presence of gender norms and how they could be operating on health-related outcomes.

A broad range of health outcomes along the life course has been shown to be associated with both sex and socioeconomic position [15]. However, few studies have considered sex, wealth, and their interactions as correlates of health outcomes during adolescence [16]. Using data from a Brazilian population-based birth cohort, we explored how the intersectionality of family income and adolescents' sex may affect health and behavioral outcomes. Although these data are disaggregated by sex, we note that disparities in health in Brazil are because of both sex-related differences due to chromosomal karyotype but most likely gender-related disparities due to culturally defined roles, responsibilities, attributes, and entitlements associated with being male or female in a given setting. Furthermore, we reflect on associations of disparities in health because of the intersection of gender and wealth.

## Methods

In 1993, a population-based cohort study was started in Pelotas, a Southern Brazilian city, with ~270,000 urban inhabitants at that time. Five maternity hospitals were visited daily, and all births from January 1 to December 31 were identified. Newborns whose families resided in the urban area of the city were examined, and their mothers were interviewed within 24 h of delivery. Information was obtained on 5,249 live births; 16 mothers refused to participate. Since then, cohort members were sought on several occasions and demographic; socioeconomic, behavioral, and lifestyle; family composition; violence; health status; and anthropometric variables were collected. Further information on the cohort procedures is given elsewhere [17,18].

In 2008, when the participants were aged 15 years, we sought to interview the full cohort. Home visits were carried out for interviews, and anthropometric measurements were taken at the research clinic. Pretested standardized questionnaires were applied by trained interviewers [17].

We used cross-sectional data from the age of 15 years follow-up. Five health and health behavioral outcome variables were studied: overweight or obesity, smoking, violence, unhappiness, and psychological symptoms. Adolescents' weight was measured to the nearest .1 kg on a digital scale (Tanita BC-418MA; Tokyo, Japan), and standing height was assessed to the nearest .1 cm using a wall-mounted stadiometer (SECA, Birmingham, UK). All measurements were performed by trained anthropometrists with the adolescent dressed in underwear and without shoes. Body mass index (BMI, kg/m^2^) was calculated and standardized by age and sex using the World Health Organization 2007 growth reference [19]. Overweight was defined as a BMI for age above +1 Z-scores [20]. This definition also includes obesity (BMI for age above +1 Z-scores) and will henceforth be referred to as overweight. The analyses were replicated considering obesity (BMI for age above +2 Z-scores) as the outcome.

Information on cigarette smoking was obtained through a confidential questionnaire from the following question: “*How many cigarettes per day did you smoke in the last 30* *days?”*. Possible answers were (1) one to five cigarettes/days, (2) 6 to 9 cigarettes/days, (3) ≥10 cigarettes/days, (4) did not smoke in the last 30 days, and (5) never smoked. We defined smokers as those adolescents who reported smoking at least 1 cigarette/day during the previous month.

Violence data were also collected through the confidential questionnaire, in which the participants answered the following question: *“Have you participated in fights at which someone was injured during the last year?"* (yes/no). This question referred to physical or verbal fights that ended with someone physically injured (by a knife, stone, or any other object that can hurt).

The adolescents were shown a happiness Smiley Face Likert Scale ranging from 1 to 7 [21,22] and asked, *"Which face shows best how you felt in the past year?"* Subjects who chose faces 1 or 2 were classified as happy, and the remainder as unhappy.

The Brazilian Portuguese version of the Strengths and Difficulties Questionnaire (SDQ), which was answered by the mothers of the participants during private interviews, was used to assess mental health. The instrument consists of 25 questions regarding the behavior of the adolescent during the 6 months before the interview. The scale is divided into five subscales (emotional symptoms, conduct problems, hyperactivity/inattention, peer relationship problems, and prosocial behavior). The total score was obtained by the sum of all subscales, ranging from 0 to 40 points [23]. Subjects with a score of 17 points or higher were considered as presenting psychological symptoms. The SDQ had been previously validated in Brazil [24]. At the 11-year visit to our cohort, the SDQ was compared with a diagnostic instrument (DAWBA, or Development and Well-Being Assessment, a diagnostic instrument for mental disorders), showing a sensitivity of 78.2%, specificity of 70.4%, positive predictive value of 48.2%, negative predictive value of 90.2%, and an area under the curve of 74.0% [25].

The five outcomes were analyzed according to biological sex, which was recorded in the perinatal assessment. We were not aware of any transgender individuals in the cohort at the time of the 15-year follow-up. Information on family income was also collected at the 15-year follow-up. This was calculated by adding the reported monthly individual income by all family members, defined as the persons living in the adolescent's household and sharing meals. This information was obtained from the adolescents' parents or caregivers. Incomes were collected as continuous variables in Brazilian reais and categorized into quintiles (Q1 the poorest and Q5 the richest). Actual income brackets by quintile were 0–380 reais; 381–600 reais; 601–1000 reais, 1,001–1,789 reais; and 1,790–40,000 reais, respectively. At the time of the interviews, one U.S. dollar was equivalent to 1.61 reais. Self-reported skin color was collected from the adolescents and coded according to the official classification of the Brazilian Institute of Geography and Statistics [26].

We described the prevalence and 95% confidence intervals of each outcome according to categories of sex (boys and girls), monthly family income (Q1 to Q5), and skin color (black, brown, and white). Fisher exact test and chi-square test for linear trends were used to compare the prevalence of the five outcomes according to sex and family income, respectively. Chi-square tests for linear trend were used to test the associations between family income and each outcome, by sex. Intersectionality was assessed through interaction tests between sex and family income (quintiles were fitted as a continuous variable) for each outcome using Poisson regression with robust variance. All analyses were performed using the software Stata 14.0 (StataCorp, College Station, TX).

The study protocols were approved by the School of Medicine Ethics Committee of the Federal University of Pelotas. Verbal consent was provided in the perinatal phase, as this was the standard practice in the country in 1993. At 15-year follow-up, full informed consent in writing was obtained both from the cohort members and from a parent or legal guardian. The respondents were assured that the data would remain confidential that their participation was voluntary, and that they could leave the study at any time, without consequences for the child or family.

## Results

At the age of 15 years, 4,320 questionnaires were answered by the adolescents and by their mothers or caretakers, 24 by caretakers only, and five by the adolescents only, totaling 4,349 contacts. Considering the 147 cohort members who are known to have died by that age, the follow-up rate was 87.5%. A total of 4,110 adolescents also attended the clinic for measurements. Supplemental Table 1 compares the full cohort with those followed up at 15 years and those included in the present analyses, according to selected variables collected in the perinatal interview. The differences were small, with a maximum of 2.2 percentage points in the maternal education variable. Adolescent boys and girls were similar in terms of family wealth, maternal education, skin color and presence of the biological father at home (Supplemental Table 2). Most mothers had up to 8 years of schooling, which corresponds to primary education in Brazil. About two in every five adolescents did not live with their biological father. At the time of the follow-up visit, 97.1% of the boys and 97.6% of the girls were still attending school.

**Figure 1:**
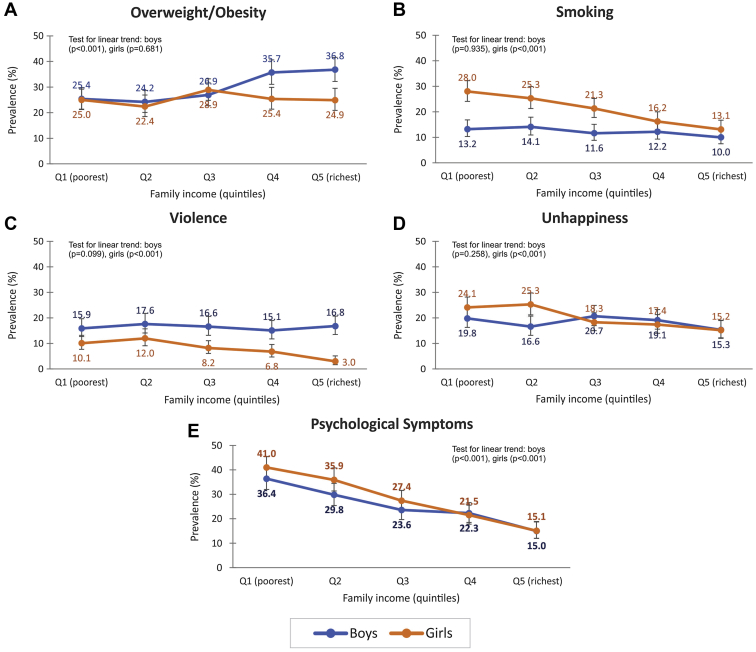
(A–E) Prevalence of adolescent outcomes according to sex and family income. Fifteen-year follow-up, 1993 Pelotas Birth Cohort.

**Supplemental Figure 1:**
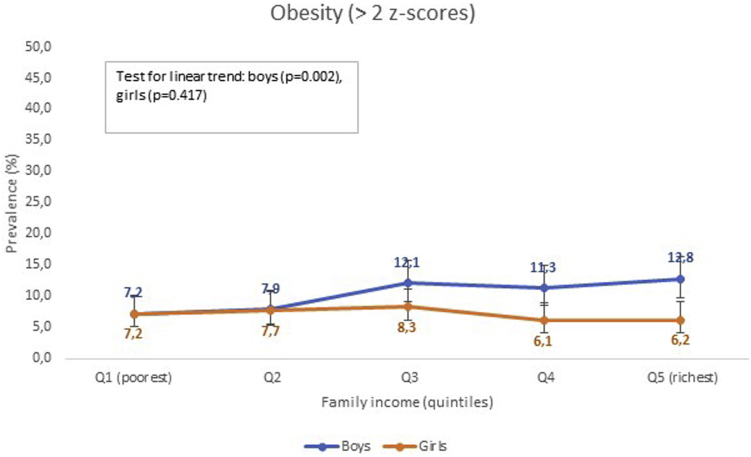
Prevalence of adolescent obesity according to sex and family income. 15-y follow-up, 1993 Pelotas birth Cohort.

Table 1 shows prevalence of the five outcomes by sex. Unhappiness was reported by similar proportions of boys and girls, but all other outcomes showed significant differences by sex. Overweight was slightly more common among boys than girls, whereas involvement with violence was twice as common among boys. On the other hand, prevalence of cigarette smoking and psychological symptoms were higher in girls than among boys. Prevalence of obesity (>2 z-scores) was 10.2% in boys and 17.2% in girls (*p* value < .001). Figure 1A–E shows outcome prevalence according to sex and monthly family income for each of the five main outcome variables. Overweight showed a direct association with family income in boys with prevalence ranging from 25.4% in the poorest to 36.8% in the richest quintile (test for linear trend in proportions: *p* < .001), whereas among girls, income differentials were not present (*p* = .681). Similar results were obtained for the prevalence of obesity (Supplemental Figure 1). Smoking prevalence was similar across family income quintiles in boys (*p* = .099), whereas a strong inverse association was found among girls, with 28% in the poorest quintile compared with 13% in the richest (*p* < .001). The patterns in violence and unhappiness were similar to that observed for cigarette smoking. Although around 16% of the boys across the five income quintiles reported violence (*p* = .935), an inverse association was observed for girls, with three times higher involvement in fights among girls in the poorest compared with the richest quintile (10% vs. 3%; *p* < .001). Unhappiness was also more common among poor than among rich girls (*p* < .001), whereas there was no social gradient for boys (*p* = .258). Psychological symptoms presented strong inverse associations with family income in both sexes. Among the poorest, prevalence of psychological symptoms was 36.4% in boys and 40.1% in girls, compared with 15.0% in the richest quintile for both sexes (*p* < .001 for boys and girls).

**Table 1:** Frequencies of five outcomes by sex of the adolescents in the 15-year follow-up, 1993 Pelotas Birth Cohort

Condition	Boys		Girls		*p* value^a^	Total
	n (%)	95% CI	n (%)	95% CI		
Overweight/obesity	2,003 (29.7)	27.7–31.6	2,098 (25.6)	23.8–27.5	.004	4,101
Smoking	2,048 (12.3)	11.0–13.8	2,158 (21.0)	19.3–22.8	<.001	4,206
Violence	2,028 (16.4)	14.9–18.1	2,149 (8.0)	6.9–9.2	<.001	4,177
Unhappiness	2,089 (18.4)	16.8–20.2	2,183 (20.1)	18.4–21.8	.176	4,272
Psychological symptoms	2,125 (25.3)	23.5–27.2	2,209 (28.3)	26.5–30.3	.014	4,334

a Fisher's exact test.

Statistical evidence of interaction between sex and family income using Poisson regression was present for overweight (β = .90; *p* = .004), smoking (β = .88; *p* = .019), violence (β = .78; *p* < .001), and unhappiness (β = 1.02; *p* = .024). Positive values of β indicate that the social gradient for boys was more marked than for girls (Supplemental Table 3).

Given the strong association between black skin color and poverty, we repeated the abovementioned analyses using skin color as the explanatory variable (Supplemental Tables 4A–E). In short, overweight and obesity tended to be more prevalent for white boys (*p* = .12) but not for girls (*p* = .66); smoking was particularly high among black or brown girls (*p* = .01); violence was particularly common among brown boys and girls and black girls (*p* = .03); unhappiness was particularly frequent among brown boys (*p* = .01); and psychological problems were less frequent among white boys and girls (*p* < .001).

## Discussion

Disaggregation of health-related variables by sex and socioeconomic position are standard components of epidemiological reports. Less attention is given to the intersectionality between these two stratifiers. Few studies have addressed how socioeconomic position intersects with sex and reflected on the influence of gender norms to shape health inequalities, particularly during adolescence [7,[27], [28], [29]].

We report on five health and health behavioral indicators among 15-year old Brazilian adolescents. These were selected to represent important dimensions of adolescents' lives that can be influenced by gender and social norms: nutrition, violence, well-being or happiness, addictive behaviors, and psychological symptoms. These conditions and behaviors, when present during adolescence, are well known to track into adulthood and contribute to major causes of morbidity and mortality throughout the life course [[30], [31], [32], [33]].

Had we ignored the intersectionality with socioeconomic position, our study would have been limited to showing higher prevalence of psychological symptoms and cigarette smoking among girls, whereas overweight and violent episodes were more frequent in boys. Of the five outcomes, only unhappiness was equally common in boys and girls. Further disaggregation by socioeconomic position revealed more complex patterns, showing significant interactions for three of the five indicators. The excess of overweight among boys is solely because of those in the higher income groups, whereas the higher rates of smoking among girls are strongly related to poverty. Violent episodes are more common among boys in all income groups, but particularly so in the better-off, as girls in the highest income bracket are seldom involved in fights. Psychological symptoms showed a strong inverse association with family income in both sexes, as was the case for unhappiness among girls, but not among boys.

Examining the 10 subgroups resulting from the combination of sex and income for each outcome, the high risk of poor adolescent girls becomes evident, with the highest prevalence in this subgroup for three of the five outcomes: smoking, unhappiness, and psychological symptoms. For overweight, the highest prevalence was among high-income boys, whereas boys from all income groups were equally likely to participate in violent fights. When we restrict the analyses to girls, inverse social gradients were present for all outcomes except overweight. In contrast, there were no social gradients for three of the five outcomes for boys, but overweight increased and psychological problems decreased with greater family income. Summing up, low socioeconomic position tends to be more strongly associated with negative outcomes for girls than for boys, which could reflect how gender norms vary according to the social context.

In-depth discussion of the determinants of adolescent health and health behaviors is beyond the scope of our analyses, but some aspects are worth noting. Regarding overweight and obesity, other studies carried out in adolescents also showed a higher prevalence in boys compared with girls [[34], [35], [36], [37]]. Most of the literature from high-income countries shows that socioeconomic position is inversely correlated with obesity in both sexes, the findings being more consistent in women than in men [27,[38], [39], [40], [41]]. The situation in Brazil is somewhat different, as studies on adults [42,43] show a direct association with income in men—similar to what we report—but an inverse association among women, which we did not document. It is possible that the social gradient for women is established later in life than at 15 years of age [44]. Gender-specific patterns related to desired body images have been shown, in the same cohort, to result in male preference to gain weight (probably related the ideal “strong body”), whereas girls are more concerned about fatness, as prevailing gender norms dictate that thinness is a desirable trait for young women [44,45].

Smoking prevalence has declined markedly in Brazil in the past couple of decades, and studies among adults show an inverse socioeconomic gradient [9,43], similar to what is observed in high-income countries [46,47]. This is consistent with the results of a national study of adolescents attending the ninth grade (mostly aged 14–15 years) in Brazil, which reported that smoking was inversely related to maternal education [48]. Therefore, our own results confirm the higher uptake of antismoking advice by wealthy than by poor adolescents, reflecting a norm that increasingly places smoking as an undesirable behavior. Our finding of higher prevalence of smoking in poor girls than boys, with divergence associated with poverty, differs from other studies, which relate higher levels of risky behaviors, including substance abuse, among boys. Smoking is widely regarded as an effective means of controlling body weight, however, and weight loss is an important concern among girls at this age, in contrast to boys who are keen to increase body weight and muscle mass [44]. Another possibility is that smoking is related to the higher prevalence of mental health problems among poor girls [49,50].

Brazil is undergoing a major crisis because of interpersonal violence. There were more than 62,000 homicides and femicides in 2018, making it the leading country in the world in absolute numbers [51]. Deaths are highly concentrated among men aged 15–24 years of low socioeconomic position and black skin color. Male sex is associated with a 12-fold increase in the risk of murder and black skin color with a fourfold increase [52]. In the present analyses, boys with brown—rather than black—skin color were most likely to be involved in violence. Globally, male adolescents are more vulnerable to outcomes related to violent behaviors than girls, either as perpetrators or victims. Their higher risk has been attributed to the social construction of masculinity linked to attitudes of violence, strength, aggression, and sexism [53,54]: “*male individuals can face particular problems because of the relation between masculine identities and risk taking*” [55]. It has also been proposed that boys are less likely to be restrained by their families, whereas the dominant stereotype for girls is related to gentle roles, which may protect them from externalizing behavior. Our findings suggest that gender roles relative to violent behavior are modulated by socioeconomic position among girls but not among boys. Girls from better-off families seem to be restrained from engaging in fights to a much greater extent than those from poor families, who are almost as likely as boys to participate in violent fights.

Mental health is an emerging priority for adolescent well-being. Suicides are a leading cause of adolescent deaths in many countries, and psychological ills—which are more common among girls than boys—contribute to almost half of their overall burden of disease [56]. As discussed below, gender norms may allow girls to report psychological problems to a greater extent than boys. We found strong inverse social gradients in reported symptoms among boys and girls. Similar gradients are reported from most adult studies in low- and middle-income countries [57], although the associations with income in these studies were not as clear cut as those with educational level or other measures of social class. One may also hypothesize whether girls in poor conditions may feel the need to assume more masculine traits, which may affect their mental health. This explanation would be consistent with the frequent involvement of poor girls in violent fights, that was documented previously.

Fewer studies, in Brazil or elsewhere, have addressed happiness or unhappiness as an outcome, compared with our four other main outcomes. A multicountry study aimed to assess social inequality in life satisfaction among adolescents (aged 11–15 years) in 41 high-income countries, mostly from Europe. Family affluence was directly associated with higher adolescent life satisfaction in nearly all countries, without significant overall differences by sex. Nevertheless, patterns by sex varied by country [58]. In Chile, adolescent girls had lower scores in several dimensions of life quality assessment than their male peers [59]. In contrast, we found similar overall results in boys and girls, although there was an inverse social gradient in prevalence among the latter.

The limitations of the study include the fact that 12.5% of the original cohort was lost to follow-up, and the data were collected in 2008–2009 and thus may not fully reflect the current status of Brazilian adolescents. The analyses by skin color (Supplemental Tables 4A–E) may lack statistical power because of relatively small number of boys and girls in the brown and black categories. Symptoms related to unhappiness may be affected by reporting bias linked to gender. Girls may be more likely to admit such symptoms compared with boys, among whom these may be regarded as weaknesses. In addition, adolescents' psychological conditions were ascertained by maternal report only to be consistent with the 11-year follow-up of the cohort. Studies from community-based samples showed that adolescents tended to rate themselves more severely than their parents [60]. We obtained information on self-reported income from the adolescents' parents or caregivers, which may be affected by reporting bias. It is important to note that sensitive information on smoking and violence was obtained through self-administered questionnaires that were later linked to the adolescents' personal information. Finally, another limitation is the lack of information regarding sexual or psychological violence or in violence that did not result in injury. The strengths of our analyses include its population basis—unlike most studies of adolescent health that are school based—and the use of locally validated instruments and standardized anthropometric measurements.

In conclusion, our analyses demonstrate the increased insight gained from studying the intersectionality of sex and socioeconomic position while applying a gender lens to better understand how gender norms affect adolescent health and behaviors in different social classes, contributing to disparities in health. Preventive strategies must take into account the intersectionality of gender and wealth and—in the particular case of Brazil—to prioritize the needs of adolescent girls from poor families.

## Supplementary data

Supplemental Table 3

Supplemental Table 4A–E
