# Supplementary material for: The Intersectionality of Gender and Wealth in Adolescent Health and Behavioral Outcomes in Brazil: The 1993 Pelotas Birth Cohort
Source: J Adolesc Health. 2020 Jan;66(1 Suppl):S51–7. doi: 10.1016/j.jadohealth.2019.08.029 (PMC6928574; doi:10.1016/j.jadohealth.2019.08.029)
Supplement: Supplemental Table 1 [file mmc1.docx]

**Supplemental Table 1. Baseline characteristics (collected at birth) for the original cohort and for the adolescents included in the analyses. 15-y follow-up, 1993 Pelotas Birth Cohort.**

|  | **Included in analyses*** | **Followed-up at 15-y** | **Original cohort** |
| --- | --- | --- | --- |
|  | **% (95%CI)** | **% (95%CI)** | **% (95%CI)** |
| **Sex** |  |  |  |
| Boys | 48.7 (47.1; 50.3) | 48.9 (47.5; 50.4) | 49.6 (48.2; 51.0) |
| Girls | 51.3 (49.7; 52.9) | 51.1 (49.6; 52.5) | 50.4 (49.0; 51.8) |
| **Monthly family income (minimum wage)** |  |  |  |
| ≤ 1 | 17.7 (16.5; 18.9) | 18.4 (17.2; 19.6) | 18.8 (17.8; 19.9) |
| 1.1 – 3 | 42.3 (40.7; 43.9) | 42.1 (40.7; 43.7) | 41.8 (40.5; 43.2) |
| 3.1 – 6 | 24.8 (23.5; 26.2) | 24.2 (22.9; 25.5) | 23.4 (22.3; 24.6) |
| 6.1 – 10 | 7.9 (7.1; 8.8) | 7.9 (7.1; 8.2) | 8.4 (7.7; 9.2) |
| > 10 | 7.3 (6.6; 8.2) | 7.4 (6.6; 8.2) | 7.5 (6.8; 8.2) |
| **Maternal education (years)** |  |  |  |
| 0 – 4 | 26.5 (25.1; 27.9) | 27.4 (26.1; 28.7) | 28.0 (26.8; 29.2) |
| 5 – 8 | 48.4 (46.8; 50.0) | 47.8 (46.3; 49.3) | 46.2 (44.9; 47.6) |
| 9 – 11 | 17.6 (16.5; 18.9) | 17.2 (16.1; 18.3) | 17.6 (16.6; 18.6) |
| ≥ 12 | 7.5 (6.7; 8.4) | 7.6 (6.9; 8.5) | 8.1 (7.4; 8.9) |
| **Birthweight (grams)** |  |  |  |
| < 2500 | 8.8 (8.0; 9.8) | 9.0 (8.2; 9.9) | 9.7 (8.9; 10.6) |
| 2500 – 2999 | 24.7 (23.3; 26.0) | 24.9 (23.6; 26.2) | 25.1 (23.9; 26.2) |
| 3000 – 3499 | 39.6 (38.1; 41.2) | 39.5 (38.1; 41.0) | 39.2 (37.9; 40.5) |
| ≥3500 | 26.9 (25.5; 28.3) | 26.6 (25.3; 27.9) | 26.0 (24.8; 27.2) |
| **Number of subjects** | 3,934 | 4,349 | 5,249 |
| *****Complete information in the five outcomes, sex and montlhy family income (compared with original cohort) | | | |
