# Supplementary material for: The Intersectionality of Gender and Wealth in Adolescent Health and Behavioral Outcomes in Brazil: The 1993 Pelotas Birth Cohort
Source: J Adolesc Health. 2020 Jan;66(1 Suppl):S51–7. doi: 10.1016/j.jadohealth.2019.08.029 (PMC6928574; doi:10.1016/j.jadohealth.2019.08.029)
Supplement: Supplemental Table 2 [file mmc2.docx]

**Supplemental Table 2. Sociodemographic characteristics of the adolescents and their parents. 15-y follow-up, 1993 Pelotas Birth Cohort.**

| Variables | Boys | Girls | Total* |
| --- | --- | --- | --- |
|  | **% (95%CI)** | **% (95%CI)** | **n** |
| Family income quintiles | | | |
| Q1 (poorest)  Q2  Q3  Q4  Q5 (richest) | 21.5 (19.8; 23.3)  18.6 (17.0; 20.4)  19.7 (18.0; 21.5)  19.2 (17.5; 20.9)  21.0 (19.3; 22.7) | 22.1 (20.4; 23.9)  18.2 (16.7; 19.8)  21.4 (19.8; 23.2)  19.3 (17.8; 21.1)  19.0 (17.4; 20.7) | 939  792  887  829  859 |
| Maternal schooling | | | |
| 0 to 4 years  5 to 8 years  9 to 11 years | 23.3 (21.5; 25.2)  40.6 (38.5; 42.8)  23.6 (21.8; 25.5) | 22.9 (21.1; 24.7)  41.7 (39.6; 43.9)  23.5 (21.7; 25.4) | 931  1,662  950 |
| 12 years or more | 12.5 (11.1; 14.0) | 11.9 (10.6; 13.4) | 492 |
| Skin color |  |  |  |
| Black | 15.0 (13.5; 16.6) | 14.4 (12.9; 15.9) | 611 |
| Brown | 18.1 (16,4; 19,8) | 19.6 (17.9; 21.3) | 784 |
| White | 66.9 (64.9; 68.9) | 66.0 (64.0; 68.0) | 2769 |
| Presence of the biological father in the home | | | |
| No  Yes | 41.1 (39.1; 43.3)  58.9 (56.7; 60.9) | 43.6 (41.6; 45.7)  56.4 (54.3; 58.4) | 1.839  2.498 |
| Total number of subjects | 2129 | 2220 | 4349 |

*Number of missing: 43 for family income. 314 for maternal schooling and 12 for presence of father in the home
