# Supplementary material for: The Intersectionality of Gender and Wealth in Adolescent Health and Behavioral Outcomes in Brazil: The 1993 Pelotas Birth Cohort
Source: J Adolesc Health. 2020 Jan;66(1 Suppl):S51–7. doi: 10.1016/j.jadohealth.2019.08.029 (PMC6928574; doi:10.1016/j.jadohealth.2019.08.029)
Supplement: Supplemental Table 3 [file mmc3.docx]

| **Conditions** | **Sex** | | | **Family income** | | | **Interaction term** | | |
| --- | --- | --- | --- | --- | --- | --- | --- | --- | --- |
|  | PR | 95% CI | p-value* | PR | 95% CI | p-value* | PR | 95% CI | p-value* |
| Overweight | 1.18 | 0.92; 1.51 | 0.18 | 1.12 | 1.07; 1.18 | <0.001 | 0.90 | 0.84; 0.97 | 0.004 |
| Smoking | 2.37 | 1.74; 3.21 | <0.001 | 0.93 | 0.86; 1.01 | 0.096 | 0.89 | 0.80; 0.98 | 0.019 |
| Violence | 0.94 | 0.65; 1.35 | 0.732 | 0.99 | 0.93; 1.07 | 0.935 | 0.79 | 0.69; 0.87 | <0.001 |
| Unhappines | 0.91 | 0.85; 0.98 | 0.012 | 1.01 | 0.99; 1.02 | 0.251 | 1.02 | 1.01; 1.04 | 0.024 |
| Psychological symptoms | 1.21 | 1.00; 1.47 | 0.051 | 0.82 | 0.78; 0.86 | <0.001 | 0.97 | 0.90; 1.04 | 0.324 |
| *Likelihood Ratio Test  PR: Prevalence Ratio  Poisson regression with robust variance. Boys are reference category. Family income in quintiles fitted as a continuous variable. | | | | | | | | | |

**Supplemental Table 3. Interactions between sex and income for the five outcomes, tested using Poisson regression with robust variance. 15-y follow-up, 1993 Pelotas Birth Cohort.**
