# Supplementary material for: The Intersectionality of Gender and Wealth in Adolescent Health and Behavioral Outcomes in Brazil: The 1993 Pelotas Birth Cohort
Source: J Adolesc Health. 2020 Jan;66(1 Suppl):S51–7. doi: 10.1016/j.jadohealth.2019.08.029 (PMC6928574; doi:10.1016/j.jadohealth.2019.08.029)
Supplement: Supplemental Table 4A–E [file mmc4.docx]

**Supplementary Tables 4a-e. Prevalence of the five outcomes according to skin color and sex. 15-y follow-up, 1993 Pelotas Birth Cohort.**

| **Table 4a. Overweight and obesity according to WHO** | | | | |
| --- | --- | --- | --- | --- |
|  | Boys | | Girls | |
|  | N | % | N | % |
| **Skin color** | p=0.124* | | p=0.664* | |
| Black | 290 | 25.5 | 302 | 26.8 |
| Brown | 349 | 28.4 | 401 | 23.9 |
| White | 1283 | 31.3 | 1323 | 25.7 |
| *Chi-squared test | | |  |  |
|  |  |  |  |  |
| **Table 4b. Smoking** | | | | |
|  | Boys | | Girls | |
|  | N | % | N | % |
| **Skin color** | p=0.070* | | p=0.001* | |
| Black | 294 | 9.9 | 302 | 24.8 |
| Brown | 355 | 15.5 | 403 | 25.8 |
| White | 1315 | 11.8 | 1380 | 18.3 |
| *Chi-squared test | | |  |  |
|  |  |  |  |  |
| **Table 4c. Violence** | | | | |
|  | Boys | | Girls | |
|  | N | % | N | % |
| **Skin color** | p=0.026* | | p=0.003* | |
| Black | 288 | 16.0 | 299 | 11.4 |
| Brown | 347 | 21.3 | 401 | 10.5 |
| White | 1308 | 15.3 | 1375 | 6.6 |
| *Chi-squared test | | |  |  |
|  |  |  |  |  |
| **Table 4d. Unhapiness** | | | | |
|  | Boys | | Girls | |
|  | N | % | N | % |
| **Skin color** | p=0.001* | | p=0.221* | |
| Black | 300 | 16.0 | 304 | 18.1 |
| Brown | 362 | 24.6 | 409 | 23.0 |
| White | 1339 | 16.6 | 1396 | 19.7 |
| *Chi-squared test | | |  |  |
|  |  |  |  |  |
| **Table 4e. Psychological problems** | | | | |
|  | Boys | | Girls | |
|  | N | % | N | % |
| **Skin color** | p<0.001* | | p<0.001* | |
| Black | 304 | 33.6 | 307 | 32.6 |
| Brown | 364 | 33.8 | 418 | 37.3 |
| White | 1356 | 21.1 | 1406 | 24.0 |
| *Chi-squared test | | |  |  |
